# Cardiac Manifestations in Children with SARS-COV-2 Infection: 1-Year Pediatric Multicenter Experience

**DOI:** 10.3390/children8080717

**Published:** 2021-08-23

**Authors:** Nicoletta Cantarutti, Virginia Battista, Rachele Adorisio, Marianna Cicenia, Claudia Campanello, Elisa Listo, Andrea Campana, Gianluca Trocchio, Fabrizio Drago

**Affiliations:** 1 Cardiology and Arrhythmias Complex Unit, Department of Pediatric Cardiology and Cardiac Surgery, Bambino Gesù Children’s Hospital, IRCCS, 00165 Rome, Italy; virginia.battista@opbg.net (V.B.); rachele.adorisio@opbg.net (R.A.); marianna.cicenia@opbg.net (M.C.); fabrizio.drago@opbg.net (F.D.); 2 Department of Neuroscience, Rehabilitation, Ophtalmology, Genetics, Maternal and Child Health (DINOGMI), Istituto Giannina Gaslini, University of Genoa, IRCCS, 16100 Genoa, Italy; claudia.campanello@gmail.com; 3 Department of Health Sciences DiSSal, University of Genoa, Ospedale-Policlinico San Martino, IRCCS, 16100 Genoa, Italy; elisa.listo@gmail.com; 4 Academic Department of Pediatrics, Bambino Gesù Children’s Hospital, IRCCS, 00165 Rome, Italy; andrea.campana@opbg.net; 5 Cardiology Unit, Istituto Giannina Gaslini, 16100 Genoa, Italy; gianlucatrocchio@gaslini.org

**Keywords:** myocarditis, cardiac involvement, children, MIS-C, SARS-COV-2, COVID-19

## Abstract

Since the spread of COVID-19, pediatric patients were initially considered less affected by SARS-COV-2, but current literature reported subsets of children with multisystem inflammatory syndrome (MIS-C). This study aims to describe the cardiac manifestation of SARS-COV-2 infection in a large cohort of children admitted to two Italian pediatric referral centers. Between March 2020 and March 2021, we performed a cardiac evaluation in 294 children (mean age 9 ± 5.9 years, male 60%) with active or previous SARS-COV-2 infection. Twenty-six showed ECG abnormalities: 63 repolarization anomalies, 13 Long QTc, five premature ventricular beats, two non-sustained ventricular tachycardia, and one atrial fibrillation. In total, 146 patients underwent cardiac biomarkers: NT-proBNP was elevated in 57, troponin in 34. An echocardiogram was performed in 98, showing 54 cardiac anomalies: 27 left-ventricular dysfunction, 42 pericarditis, 16 coronaritis. MIS-C was documented in 46 patients (mean age 9 ± 4.8 years, male 61%) with cardiac manifestations in 97.8%: 27 ventricular dysfunctions, 32 pericarditis, 15 coronaritis, 3 arrhythmias. All patients recovered, and during follow-up, no cardiac anomalies were recorded. Our experience showed that cardiac involvement is not rare in children with SARS-COV-2, and occurred in almost all patients with MIS-C. However, patients’ recovery is satisfactory and no additional events were reported during FU.

## 1. Introduction

Since the spread of COVID-19 worldwide, children have been initially considered less commonly affected by SARS-COV-2. Although largely with mild symptoms, these latter in children may vary greatly, including: fever, cough, gastrointestinal manifestations, polymorphic rash, conjunctivitis and poor feeding, especially in infants.

In the recent literature, a subset of pediatric patients with a severe systemic hyper-inflammation linked with SARS-COV-2 infection has been reported [1,2]. The increasing number of reported cases led to a health advisory from the Royal College of Pediatrics and Child Health (RCPCH), the Centers for Disease Control and Prevention (CDC), and the World Health Organization (WHO), which identified these cases as a novel condition named multisystem inflammatory syndrome in children (MIS-C) [3,4,5]. These patients showed elevated inflammatory markers and cytokine storms with a multi-organs involvement including cardiac manifestations, ranging from pericarditis, myocardial dysfunction, systemic hyper-inflammation/vasodilation, Kawasaki-like disease to arrhythmias [6,7,8,9,10,11]. Management of this condition is based on expert consensus and includes immunomodulatory agents, anticoagulation and cardiac support.

The aim of this study is to describe a pediatric multicenter experience on cardiac manifestations due to SARS-COV-2 infection in children after one year of pandemic.

## 2. Materials and Methods

This is a multicenter retrospective study. We included in the study all consecutive symptomatic pediatric patients with active or previous SARS-COV-2 infection confirmed by nose and pharyngeal swab SARS-COV-2 nucleic acid test or antibodies detection, admitted to Bambino Gesù Children’s Hospital (BGCH) and Giannina Gaslini Institute (GGI) COVID centers. These “Referral Centers” were created for the SARS-COV-2 pandemic for the Lazio and Liguria region in March 2020.

The clinical records were reviewed in order to identify all patients with COVID-19 or MIS-C related cardiac involvement. Data including demographic information, contact history, previous history, clinical symptoms, treatment, imaging and laboratory findings during the hospital stay were recorded.

The laboratory tests for SARS-COV-2 detection were performed in accordance to the guidelines and in laboratories identified by Italian Health Ministry. Microbiological data accounted of nasopharyngeal, stool, urine, bone marrow and conjunctival swab for SARS-COV-2 detection by the mean Real-Time Polymerase Chain Reaction (RT-PCR) and antibodies for SARS-COV-2 detection.

Definition of MIS-C included presence of fever, laboratory evidence of inflammation, and multisystem organ involvement without alternative plausible diagnoses, as well as evidence of COVID-19 infection or recent exposure to a COVID-19 case. Diagnosis of MIS-C was confirmed based on WHO criteria [5].

For patients with MIS-C, the microbiological workout included also blood viral panel of Human Immunodeficiency virus, Hepatitis B virus, Hepatitis C virus, Cytomegalovirus, Epstein-Barr virus, Parvovirus B19, Herpes simplex virus 1 and 2, Human Herpes virus 6 and adenovirus.

All patients were evaluated by laboratory blood test, including multi-organ function, infection rates and inflammatory markers, ECG and chest X-ray. In patients with ECG anomalies or with signs of hyper-inflammation, echocardiography and cardiac biomarkers, including NT-proBNP and troponin were performed. Patients with cardiac involvement had continuous electrocardiographic monitoring and were daily revaluated with ECG and echocardiography during acute event. Cardiac biomarkers with troponin were repeated every 6 h if elevated; NT-proBNP once a day if clinical condition was stable. In case of cardiac rhythm anomalies, 24-hours ECG monitoring was performed.

Out of acute event, cardiac examination, ECG, echocardiography, 24-hours ECG monitoring and cardiac biomarkers were performed before discharge.

Patients with acute cardiac involvement were regularly evaluated in follow-up (FU) at 1 month, 3 months, 6 months and one year from acute event.

Statistical analysis was performed by SPSS Statistics 21 (IBM Corporation, Armonk, NY, USA). Categorical variables are expressed as absolute numbers or percentages. Chi-squared test was used to analyze variables. *p*-value was considered significant when less than 0.05.

This study has been approved by the institutional committees of Bambino Gesù Children’s Hospital IRCCS and Giannina Gaslini Institute IRCCS within the Research of the Ministry of Health (RCR-2020-23670065). Written informed consent was signed by parents or legal guardians for invasive or not routine investigations. All investigations were conducted according to principles expressed in the Declaration of Helsinki.

## 3. Results

Between March 2020 and March 2021, cardiac evaluation was performed in 294 pediatric patients with active or previous SARS-COV-2 infection confirmed by nose and pharyngeal swab SARS-COV-2 nucleic acid test or antibodies detection, admitted to BGCH and GGI pediatric COVID centers.

**Figure 1:**
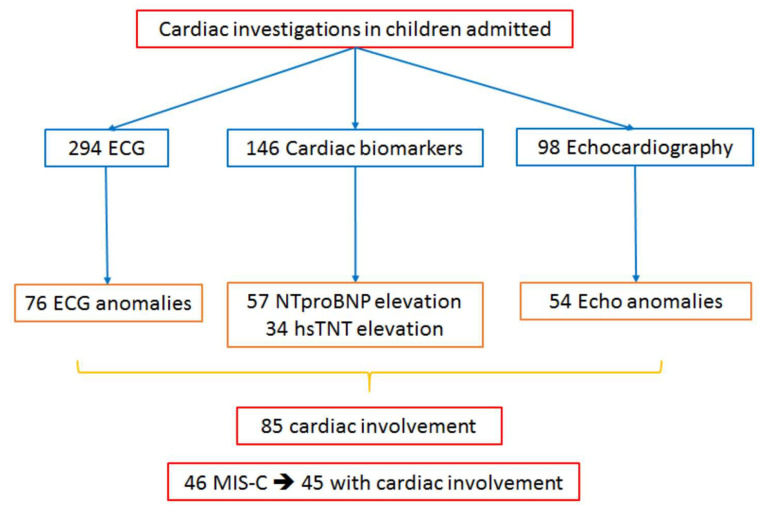
Flow chart of cardiac investigations of children admitted with SARS-COV-2 infection.

Among the 294 children evaluated, 85 (mean age 9 ± 5.9 years, male 60%) showed different patterns of cardiac manifestations ranging from isolated ECG anomalies to myocardial involvement with severe systolic dysfunction (Figure 1, Table 1).

Cardiac symptoms ranged from poor feeding, chest pain, asthenia or hypotension, particularly in MIS-C.

In particular, 76 patients (26%) showed ECG abnormalities. Among them: 63 patients showed ventricular repolarization (VR) abnormalities with flat or negative T-waves in infero-lateral leads (normal-for-age changes in V1-V3 were not considered), 13 prolonged QTc interval and bradycardia, two of them developed non-sustained ventricular tachycardia (NSVT), five premature ventricular beats (PVBs) and one atrial fibrillation (AF) (Table 2).

Cardiac biomarkers were investigated in 146 patients. NT-proBNP was elevated in 57 (19%) (mean value 5057 ± 8640 pg/mL) and Troponin in 34 patients (12%) (mean value 94 ± 206 pg/mL).

Due to ECG anomalies or elevated cardiac biomarkers, 98 patients underwent echocardiography. Among these, 54 patients (18%) showed cardiac anomalies including: left ventricular (LV) systolic dysfunction in 27 with a mean ejection fraction (EF) of 42%, pericardial effusion in 42 and coronary artery hyper-echogenicity or mild dilation (with z-scores 2–2.5) in 16 (Table 3).

MIS-C occurred in 46 patients (mean age 9 ± 4.8 years, 61% male). Among them, 45 (97.8%) showed cardiac manifestations: LV systolic dysfunction in 27, pericardial involvement in 32, coronary disease in 15, arrhythmias in three, prolonged QTc in eight. Twenty-seven (59%) out of 46 patients with MISC were admitted in pediatric intensive care unit (PICU) and 41% of them needed inotropic support.

Among all patients studied, gastrointestinal involvement was predominant (48%): vomiting, diarrhea or poor feeding (particularly in infants) were frequent symptoms at the admission. Patients with MIS-C showed gastrointestinal symptoms in 72% of cases (33 patients).

Based on the type and the severity of cardiac involvement, patients with MISC were treated by intravenous immunoglobulin (2 gr/kg) in case of coronary or pericardial disease; by magnesium infusion (50 mg/kg i.v.) in case of long QT with hyperkinetic arrhythmias, by inotropes such as milrinone 0.5 mcg/kg/min i.v. in patients with systolic dysfunction, in association to antiplatelet (aspirin 3 mg/kg) and/or anticoagulation treatment (low molecular weight heparin 100 UI/kg s.c.) and anti-inflammatory therapies including intravenous corticosteroid alone (methylprednisolone 2 mg/kg i.v.) or in addition to anakinra (2 mg/kg s.c.) in most severe patients.

All patients recovered after therapy and no deaths for Sar-Cov-2 infection nor MIS-C were recorded, only one oncological patient died, but not for infection or cardiac reasons.

Patients with myocardial damage were discharged with corticosteroids therapy (prednisone 1 mg/kg, gradually reduced to discontinuation), but without specific cardiac drugs.

All patients with cardiac involvement are still regularly evaluated in FU: almost 9.4% of patients have reached one-year of FU, 22.4% of patients had a 6-month FU, 49.4% of patients reached 3-month of FU and 18.8% had 1-month of FU.

At the time of FU, no complications including ECG or echocardiography anomalies or increased cardiac biomarkers or cardiac death were recorded. Only one death was documented, but not due to infection or cardiac reasons.

## 4. Discussion

This is the first pediatric study describing cardiac manifestations in all-comer children with SARS-COV-2 infection and not only in MIS-C patients.

MIS-C is a new syndrome related to SARS-COV-2 infection, characterized by hyper-inflammation signs and multi-organ dysfunction. Some recent studies suggest that MIS-C is caused by a post-infectious inflammatory syndrome associated with elevation in all cytokines and markers of recent T-cell activation occurring despite a strong and specific humoral response to SARS-COV-2 [12].

Cardiac involvement is frequently described in children with MIS-C, ranging from myo-pericardial inflammation, coronary dilation or aneurysm and arrhythmias.

Cardiac support, immunomodulation, and antiplatelet/anticoagulation treatments are part of the management of acute MIS-C [2].

However, to the best of our knowledge even if cardiac involvement was already described in patients with MIS-C, no study reported the incidence of cardiac manifestation in all children admitted for SARS-COV-2 infection.

### 4.1. Pericarditis

Data about the spectrum of clinical presentation of COVID-19 infection in children is limited, and frequency of pericardial manifestation is still unknown. The mechanism of pericarditis in COVID-19 is probably due to the inflammatory process and the subsequent cytotoxic and immune-mediated effects related to SARS-COV-2 infection. In the literature, one case of acute pediatric pericarditis presenting with pericardial tamponade [13] and two cases of adolescents with acute pericardial effusion [14] have been already reported. In our cohort, 42 patients showed pericardial involvement, representing 50% of all patients and 66% of MIS-C.

### 4.2. Myocarditis and Myocardial Dysfunction

Myocardial involvement with ventricular systolic dysfunction until now has been reported in a relatively large number of pediatric patients with MIS-C. In the first MIS-C case-series, LV dysfunction was described in a different percentage of children, ranging from 35 to 100% of patients [15,16,17,18,19,20,21,22,23,24,25,26,27,28,29,30,31,32,33]. Some reports described patients with shock or severe LV dysfunction requiring mechanical ventilation, inotropic support and extracorporeal membrane oxygenation (ECMO) [21,22,23,24,25]. A high proportion of elevated troponin and BNP or NT-proBNP levels were noticed. All patients successfully recovered, and no deaths have been recorded.

In our cohort of patients, LV systolic dysfunction was detected in 32% of all patients and in 59% of patients with MIS-C. NT-proBNP was elevated in 19% and troponin level was high in 12% as marker of cardiac damage. No cases of cardiogenic shock or ECMO support were recorded and all patients had a full recovery of LV function prior to discharge.

The mechanism of myocardial dysfunction in MIS-C has not been fully understood and probably different pathophysiological mechanisms may be responsible. Possible causes include acute myocarditis, post-viral immunological reaction and systemic inflammatory response syndrome.

### 4.3. Electrocardiographic Alterations and Arrhythmias

Arrhythmic manifestations are described in 7 to 60% of patients with MIS-C with different patterns of presentation. The most frequently reported ECG anomalies were non-specific and included VR anomalies, QTc prolongation, and premature atrial or ventricular beats. First- and second-degree atrioventricular (AV) blocks were reported in one study and atrial fibrillation in two [16,29]. Comparing our study data to the literature, we have found a low percentage of rhythm abnormalities: 3% of all our cohort and 7% of patients with MIS-C. In our cohort, QTc prolongation was documented in 17% of patients with MIS-C, no cases of AV block were recorded, while a case of AF was documented. In our study population ventricular arrhythmias included PVBs in five patients and NSVT in two, whilst we had no cases of sustained arrhythmias requiring emergency treatment and ECMO support for hemodynamic deterioration, as previously reported [15,29].

### 4.4. Coronary Involvement

MIS-C overlaps for many aspects with Kawasaki disease (KD), characterized by vasculitis of medium vessels on inflammatory basis. Some studies reported an increase of Kawasaki-like disease during COVID-19 pandemic in patients positive for SARS-COV-2 IgG antibodies. When these cases were compared with classical KD, they resulted to be older, more likely to present in shock, to have more cardiac involvement and to show more elevated levels of cardiac biomarkers [18,29]. KD can have a number of long-term sequelae, including persistent coronary aneurysms, occurring in 20–25% of untreated children [34,35,36,37]. Coronary dilation or aneurysms have been reported in up to 25% of MIS-C patients, suggesting a pathophysiologic similarity with KD [15,18,21,22,23,28,29,30,31,32]. In most cases mild coronary artery dilation was described (with z-scores 2–2.5), considering that in the acute phase it may be related to coronary vasodilation in the setting of fever and inflammation. However, there have also been reports of large and giant coronary artery aneurysms [15,29], with progression during FU [15,18,28,29].

In our cohort, coronary artery involvement was found in 5% of all patients and 33% of MIS-C patients. However, no case of large or giant aneurysms or coronary thrombosis were noticed during acute event or recorded during follow-up.

## 5. Conclusions

This multicenter experience shows that cardiac complications occur in about 30% of children with SARS-COV-2 infection and in almost all those with MIS-C, generally starting with gastrointestinal symptoms in more than half of them.

The most frequent cardiac manifestation is pericardial effusion followed by LV systolic dysfunction. Whilst, coronary abnormalities occurred in few patients and in about one-third of children with MIS-C. Isolated VR abnormalities are widespread, especially in MIS-C patients, but complex arrhythmias are rare. Few patients required initial PICU admission, but all cardiac manifestations were no more detectable at mid-term FU.

This is the first pediatric study describing cardiac manifestations in all-comer children with SARS-COV-2 infection and not only in MIS-C patients. In our cohort, we found a high prevalence of cardiac involvement paired with excellent outcomes.

Early identification of the population at risk is necessary for early treatment and proper management of these patients. Undoubtedly, such kind of approach has been proven effective in avoiding more severe expressions of cardiac involvement and determining patients’ good prognosis. More studies and long-term follow-up are required to identify eventual late clinical phenomena and better understand long-term prognosis.

## 6. Study Limitations

The main limitation of our study is its retrospective nature, the mid-term follow-up and the relatively limited number of patients. The use of Cardiac Magnetic Resonance (CMR) for the evaluation of these patients, particularly MIS-C patients, would be useful to better understand the nature of myocardial involvement, to define the extension of cardiac damage, to stratify patients at high risk of severe forms or complications, and finally to identify long term scar or sequalae. CMR was not initially performed because of the rapid recovery of myocardial dysfunction after treatment and the requiring of general anesthesia in pediatric patients. Further multicenter studies and long-term follow-up are required to better understand the incidence and consequence of cardiac involvement due to Sars-Cov-2 infection in children.

## Figures and Tables

**Table 1:** Cardiac anomalies in children with SARS-COV-2 infection (No MIS-C vs. MIS-C). MIS-C: multisystem inflammatory syndrome-children.

Children COVID-19	N.	%	No MIS-C	%	MIS-C	%	*p*-Value
Cardiac investigation	294		248		46		0.45
Cardiac involvement	85	29%	40	16%	45	98%	<0.0001
ECG anomalies	76	26%	44	18%	32	70%	<0.0001
Echocardiography anomalies	54	18%	12	5%	42	91%	<0.0001
Cardiac biomarkers elevation:							
NTproBNP	57	19%	19	8%	38	83%	<0.0001
hsTNT	34	12%	5	2%	29	63%	<0.0001

**Table 2:** Classification of ECG anomalies (76 patients).

ECG Anomalies	N.	%
Repolarization abnormalities	63	83%
QTc prolongation and bradycardia	13	17%
NSVT	2	3%
PVBs	5	7%
SVT	1	1%

NSVT: Non-sustained ventricular tachycardia; PVBs: premature ventricular beats, SVT: supraventricular tachycardia.

**Table 3:** Classification of echocardiography anomalies (54 patients).

Echocardiography Anomalies	N.	%
Left ventricle systolic dysfunction	27	50%
Pericardial effusion	42	78%
Coronary involvement	16	30%

## Data Availability

The data presented in this study are available on request from the corresponding author. The data are not publicly available due to Institutional and Research policies.
